# Changes in very older person admissions to an ICU over a decade

**DOI:** 10.1186/cc9888

**Published:** 2011-03-11

**Authors:** J Connelly, E Dawe, H Jones

**Affiliations:** 1 Morriston Hospital, Swansea, UK

## Introduction

The UK population is getting older and consequently our attitudes, together with the number of very older person admissions to critical care, may be changing [1]. We therefore reviewed admissions aged 80 or over (very older people) to our ICU and compared this with 10 years ago.

## Methods

Retrospective data collection was completed for all patients admitted to our ICU for a 12-month period starting in August 2009, and a comparable 12-month period starting August 1999. Data were retrieved from an electronic database of ICU admissions.

## Results

The number of very older patients admitted for the 1999 period was 87 out of a total 702 (12.4%) and for the 2009 period was 156 out of a total 1,071 (14.6%). There was a marked increase in emergency medical (from 24% to 47%) and emergency surgical (from 31% to 41%) admissions in the 10 years. This was in contrast to elective surgical admissions, which have reduced from 42% to 12%. The mean ICU admission APACHE II score for patients over 80 years old decreased from 21.2 to 18.6. The ICU and hospital mortality for the very older people is summarised in Table 1. The ICU mortality for this age group increased from 29% to 33% but the hospital mortality was unchanged at 44%.

**Table 1:** Mortality of very older patients by specialty at ICU/hospital discharge

	Medical (%)	Emergency surgical (%)	Elective surgical (%)	Overall (%)
1999 to 2000	33/52	41/48	18/36	29/44
2009 to 2010	38/48	34/47	11/16	33/44

Data presented as ICU/hospital mortality (%).

## Conclusions

Changes in population demographics are reflected in our critical care by an increase in the number of very older person admissions. The ICU mortality was higher in this group compared with 10 years ago. One possible explanation is the marked increase in emergency admissions. This may reflect an increased willingness to refer the very older patient for critical care support.
